# Effect of green leafy vegetables powder on anaemia and vitamin-A status of Ghanaian school children

**DOI:** 10.1186/s40795-018-0235-x

**Published:** 2018-06-08

**Authors:** Godfred Egbi, Samuel Gbogbo, George Ekow Mensah, Mary Glover-Amengor, Matilda Steiner-Asiedu

**Affiliations:** 1 Noguchi Memorial Institute for Medical Research, College of Health Sciences, University of Ghana, P.O. Box LG 581, Legon, Accra, Ghana; 2 Department of Nutrition and Food Science, College of Applied and Basic Sciences, University of Ghana, P.O. Box LG 134, Legon, Accra, Ghana; 3 CSIR, Food Research Institute, P.O Box m 20, Accra, Ghana

**Keywords:** Anaemia, Green leafy vegetable, Powder, School children, Vitamin-A, Deficiency, Prevalence

## Abstract

**Background:**

Nutritional anaemia and vitamin-A deficiency are public health issues confronting Ghanaian children. Their adverse effects are likely pronounced during the dry season when green leafy vegetables, rich-sources of iron and provitamin-A are scarce. This study assessed the effect of dried green leafy vegetables on anaemia and vitamin-A status of Ghanaian school children.

**Method:**

This was 3 months pretest, posttest nutrition intervention study. Children 4–9 years were randomized to receive or not receive supplement. High Performance Liquid Chromatography and Haemocue hemoglobinometer were used to determine vitamin-A and haemoglobin concentrations respectively. Malaria-parasitaemia and helminthes were examined by Giemsa-staining and Kato-Katz respectively. Nutritional status was assessed by anthropometry. Student’s t-test was used to establish significant differences between groups.

**Results:**

At baseline, the mean haemoglobin concentrations of control and supplemental were 116.9 ± 9.9 g/l and 117.6 ± 12.7 g/l respectively. At end-line, it was 121.9 ± 13.5 g/l for supplemental and 113.4 ± 8.5 g/l for control, significant at *p* = 0.001. At baseline prevalence of anaemia was 37.3 and 41.5% in control and supplemental respectively. At end-line it was 33.3% in supplemental against 57.5% in control, significant at *p* = 0.024. At baseline mean retinol concentrations were 16.79 ± 8.74 μg/dl and 16.97 ± 7.74 μg/dl for control and supplemental respectively. Mean retinol concentrations for control and supplemental were 24.35 ± 5.50 μg/dl and 26.96 ± 6.86 μg/dl respectively at end-line. At end-line 60% of control against 64.0% of supplemental had low vitamin-A status. At end-line, anaemic-control had mean retinol concentration of 23.78 ± 5.23 μg/dl and anaemic-supplemental had 27.46 ± 7.28 μg/dl. Prevalence of low vitamin-A status was 64.3 and 84.2% in anaemic-control and anaemic-supplemental respectively at baseline but it became 23.1 and 21.1% respectively, at end-line. The mean haemoglobin concentrations of anaemic-control and supplemental were 105.7 ± 7.5 g/l and 113.6 ± 13.6 g/l respectively at end-line. The change in prevalence of anaemia between the anaemic groups was 12.2%, significant at *p* = 0.042.

**Conclusion:**

Consumption of green leafy vegetables powder increased mean haemoglobin and retinol concentrations of the study participants. It had the potential to minimize prevalence of anaemia and low vitamin-A status of study participants.

## Background

Anaemia affects averagely 800 million children and women worldwide [1]. In Sub Saharan Africa, about 83.5 million people are anaemic [2]. Anaemia and vitamin A deficiency are persistent nutritional problems of public health interest confronting Ghanaian children [3–6]. In Ghana, anaemia was the fourth cause of hospital admissions and the second factor contributing to mortality after a review of the disease profile and pathology reports in some hospitals [7]. Anaemia impairs children’s mental, physical, social development and it is related to their poor academic performance [8]. The long effect of anaemia due to iron deficiency on Intelligence Quotient (IQ) is 1.73 points lower for every 10 g/l decrease in haemoglobin levels at the early stage of life [9]. Anaemia is caused by multiple factors: nutritional and non-nutritional factors [10–12]. Nutritional anaemia is attributable to iron, vitamin A, folate, vitamin B_12_, ascorbic acid and zinc deficiencies [13]. These nutrients deficiencies may be due to inadequate dietary intakes and poor bioavailability of these micronutrients. Poor bioavailability is the inhibitory effect of anti-nutritional factors such as polyphenols, tannins and phytates [13]. Inflammation, infection and genetic disorders (thalassaemia, sickle cell disease) which affect erythrocytes are other factors known to cause anaemia [14].

Vitamin-A deficiency contributes to nutritional anaemia. Linkage existed between vitamin-A deficiency and general anaemia such that anaemia prevalence reduced repeatedly during simultaneous vitamin-A, and iron supplementation [15, 16]. It has also been shown that vitamin-A, and iron supplementation had positive impact on measures of vitamin-A and anaemia status in older children [15, 17]. Vitamin-A deficiency is the most essential causes of avoidable childhood blindness and is a major contributor to morbidity and mortality from infections [18]. There have been attempts and there are still strategies to minimize the burden of anaemia among Ghanaian children. Most of these strategies include food fortification, dietary modification and diversification, access to and use of mosquito treated bed nets and nutrition education programs. Diets in developing countries are known to lack a wide range of micronutrients [19]. In tropical Africa where the daily diet is mainly composed of starchy staples**,** green leafy vegetables (GLVs) are the cheapest and most readily available source of micronutrients [20]. Several studies have been done on the nutritional value of many GLVs and it has been established that their consumption on regular basis may result in the reduction of micronutrients deficiencies leading to improvement in the nutrition and wellbeing of humans. It is reported that nearly 1000 edible vegetables exist in sub-Saharan Africa [21]. In Ghana, there are two seasons; the rainy and dry seasons. Green leafy vegetables are only available, accessible and affordable to most households during the rainy season. During the dry season, most rural household members have inadequate dietary intakes of micronutrients such as iron, zinc and pro vitamin-A (beta-carotene from GLVs) are virtually absent. They would be unable to meet their Recommended Dietary Allowances (RDA’s) for micronutrients such as iron, folic acid, vitamin-A and zinc [22] which may exacerbate the problem of nutritional anaemia among Ghanaian school children. One strategy would be to process GLVs into products like powders that can be well preserved to ensure good keeping quality so that they can be available, accessible and affordable to most households in the dry season. Sun drying and oven (mechanical) drying are two options. Sun drying is the least expensive and most accessible way of food preservation in low income countries. Green leafy vegetable powder of *Solanum macrocarpon* (eggplant) and *Amaranthus cruentus* (amaranthus) are rich sources of iron, zinc and beta-carotene [20, 23, 24] so could contribute to the reduction of anaemia and Vitamin-A deficiency. Hence this study seeks to assess the effect of consumption of Composite Green Leafy Vegetable Powder (CGLVP) of *Solanum macrocarpon* and *Amaranthus cruentus* on anaemia and vitamin-A status of Ghanaian school children.

## Methods

The study was a pretest posttest design. The participants were school children 4–9 years old. Inclusion and exclusion criteria were school children 4–9 years, not severely anaemic, not on any nutrient supplement and participating regularly in the Ghana Government School Feeding Program. Data on background characteristics (sex, age, occupation, education and monthly income) were obtained from parents of the study participants through interviews using combined open-ended and semi-structured questionnaires. Height and weight measurements were done according to standard procedures [25]. Duplicate weight measurements were taken to the nearest 0.1 kg using an electronic bathroom scale (Precision Health Scale UC-300 from A and D Company Limited, Higashi-Ikebukuro, Toshima-Ku, Tokyo, Japan). Each child’s height was taken with a Plastic stadiometer in a standing position. Heights were taken in duplicates to the nearest 0.1 cm. The mean of two readings was considered as the actual value for each participant.

**Fig. 1:**
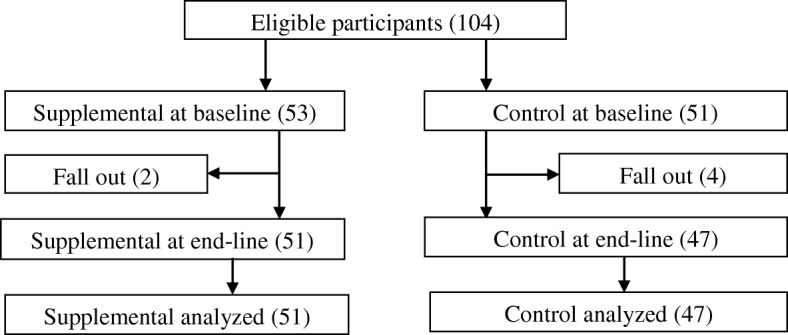
Consort diagram of the study

Five millilitres of fasting venous blood was obtained from each participant by a phlebotomist into Eppendorf tubes without anticoagulants in the morning before breakfast. The blood samples were sent to the clinical laboratory of the Volta Regional Hospital (Trafalga), centrifuged and serum aliquots pipetted into Eppendorf tubes and stored below -80^o^ C until they were analyzed for vitamin-A. Parents helped collect 3 g (teaspoon size) of fresh stool sample from their wards participating in the study early in the morning before breakfast. The stool samples were immediately transported on ice to the microbiology-parasitology laboratory at Trafalga and examined for the presence of soil-transmitted helminthes with the Kato-Katz technique [26]. Prior to the distribution of stool containers, parents of participating children were educated on all the necessary safety measures and taken through the appropriate way to collect stool samples. Haemoglobin concentrations were assessed immediately in the field using a Hemocue Hemoglobinometer (Hemocue AB, Angelhom, Sweden). Haemoglobin values were taken to the nearest 0.1 g/l. Malaria parasitemia was examined in thin and thick blood film slides of participants using the Giemsa staining technique [26]. Children with severe anaemia (haemoglobin < 85 g/L) were excluded from the study but referred by the medical officer on the study team to the Ho municipal hospital for treatment. Children found with malaria parasitaemia were referred by the medical officer on the study team to the Ho municipal hospital to seek treatment. They were recruited into the study as they met the study inclusion and exclusion criteria.

Serum vitamin A concentration for each participant was determined by HPLC using a modified procedure of Noguchi Memorial Institute for Medical Research (NMIMR) protocol for vitamin A analysis in serum [27]. Initially 1 mg/ml standard retinol stock solution was prepared. A standard working solution of 1 μg/ml retinol standard was prepared from the stock solution. Serial dilutions of 0.5 μg/ml, 0.25 μg/ml, 0.125 μg/ml and 0.0625 μg/ml standard retinol solutions were made from 1.0 μg/ml of the standard working solution. Aliquots of 240 μl of respective standard solutions were injected into the HPLC system and their peak areas determined. The standard curve was established by plotting mean peak area against respective mean standard concentration. Frozen serum samples of the study participants were completely thawed for 60 s. Duplicate aliquots of 120 μl serum for every study participant was pipetted into 1.5 ml Eppendorf tubes and 120 μl of methanol added. The mixture was vortexed for 30 s to denature all protein materials present in the serum sample. Five hundred microliters of hexane was added and the resultant mixture was vortexed for 120 s to solubilize all the fat soluble components. Two hundred and fifty microliters of supernatant was pipetted into a clean 1.5 ml Eppendorf tube and evaporated slowly and carefully under nitrogen gas to dryness. The residue was reconstituted with 120 μl of methanol by vortexing for 20 s. The reconstituted extracted sample was injected into an HPLC system and the peak area recorded. Duplicate determinations of peak areas were made on each serum sample and the average used to determine the serum retinol concentration in a sample. The HPLC was calibrated such that the retinol component eluted at 4.5 min after injection. The mobile phase was methanol (HPLC grade) and the stationary phase is Purospher® STAR RP-18 endcapped (5 μm) Hibar® RT 250–4,6 HPLC column. The operation wavelength was 350 nm (Figure 1).

### Composite green leafy vegetable powder preparation

Leafy vegetables of *Solanum macrocarpon* (eggplant) and *Amaranthus cruentus* (amaranthus) leaves were purchased from farmers engaged in urban vegetables market gardening. Each vegetable sample was washed in clean water, 1% saline for 3 min, rinsed with clean tap water and dried in a locally manufactured mechanical oven at 45 °C for 10 h. The dried leaves were milled into fine powder with a blender. One hundred and sixty grams of powdered *Solanum macrocarpon* and same quantity of *Amaranthus cruentus* were weighed and mixed thoroughly with a cake mixer. Two hundred and thirty grams of the mixed product, (CGLVP) was packaged in airtight plastic (polythene) bags and stored in cardboard boxes.

### Intervention feeding

Beans and tomato stews and groundnut soup each was prepared with, tomato paste (200 g), granulated pepper (15 g), onion paste (35 g), smoked anchovies powder (100 g) and iodized salt (40 g) with or without 230 g of Composite Green Leafy Vegetable Powder (CGLVP). Groundnut oil (400 g) was used to prepare either beans or tomato stew and Groundnut paste (400 g) was used to prepare the groundnut soup. Each participant was served 50 g of tomato stew with or without CGLVP thrice a week, 100 g of beans stew with or without CGLVP and 95 g of groundnut soup with or without CGLVP once a week for a period of 3 months. Chemical analysis established that tomato stew, beans stew and groundnut soup alone provided 3.0 ± 0.02 mg, 5.15 ± 1.01 mg and 2.8 ± 0.2 mg iron respectively to each participant in the control group. Tomato + CGLVP stew, beans + CGLVP stew and groundnut + CGLVP soup provided 9.7 ± 0.1 mg, 14.5 ± 1.1 mg and 6.9 ± 0.1 mg iron respectively to each study participant in the experimental group. The supplemental stews and soup (Tomato + CGLVP stew, beans + CGLVP stew and groundnut + CGLVP soup) provided an average intake of 498.1 μg beta-carotene (41.5 RE)/day against 275 μg - 445 μg beta-carotene (22.9 – 30RE) which is the estimated average requirement of pro vitamin A for 4–9 years children [22]. At the end of 3 months intervention feeding, blood and stool samples were collected, examined and analyzed for the various factors just as done at baseline.

### Data analysis

Data at baseline and at the end of the study were entered into Epi Info Version 7 software, cleaned and exported to SPSS version 22.0 for analysis. Children were classified as anaemic with haemoglobin concentration below 110 g/l and 115 g/l for children 5–59 months and 5–11 years respectively [28]. The cut off used to identify participants with low vitamin-A status was serum retinol concentration < 20 μg/dl. Summary data were presented with descriptive statistics as means plus standard deviations, frequencies and percentages. Data comparison within each group was done for differences in anthropometric measures, haematological and biochemical variables using paired t-tests. Between groups significant or non-significant differences for continuous variables were carried out using independent t-test. Chi-square test was used to establish differences in percentages or proportions for categorical variables between groups. Binary logistic regression analysis was done to identify factors related to anaemia among the study participants. Significant differences were set at *p* < 0.05 level.

## Results

The characteristics of the study participants are shown in Table 1. The mean age was between 6.7–7.3 years. At baseline, the prevalence of malaria parasitaemia was comparatively similar in the range of 34.0–37.0%. It was 40.4% in the controls and 39.6% in the experimental (supplemental) at the end of the study (end-line). Hookworm infestation was 1.0% at baseline and absent at end-line. There was high patronage of treated mosquito net use in the range of 86–91%. Majority of the study participants and their household members (83–88%) were beneficiaries of National Health Insurance Scheme (NHIS). Most parents (or guardians), 94–96% had formal education (Table 1). As shown in Table 2, the mean haemoglobin concentration of the supplemental group was 121.9 ± 13.5 g/l and that of the control was 113.4 ± 8.5 g/l at the end of the study. The mean value for the supplemental group was significantly different from that of the control group, *p* < 0.05. The prevalence of anaemia among the control group (57.5%) was higher than the prevalence among the supplemental group (33.3%), Table 2. The mean serum retinol concentration for the control and the supplemental groups at the start of the study were 16.79 ± 8.74 μg/dl and 16.97 ± 7.74 μg/dl respectively (Table 3**)**. The mean serum concentration increased to 24.35 ± 5.50 μg/dl in the control group and to 26.96 ± 6.86 μg/dl in the supplemental group. The results (Table 3) show that at baseline 60.0 and 64.0% of the participants in the control and supplemental groups respectively had low vitamin A status. The percentage of participants with low vitamin A status declined to 18.2% in the control and 18.8% in the supplemental group at the end-line. The percentage of participants with normal vitamin A status in the control was 40.0% at baseline. It was 34.0% in the supplemental group. It increased to 84.0% in the control and to 87.0% in the supplemental group at the end-line, Table 3. Table 4 shows the post vitamin A and anaemia status of anaemic participants before the start of the nutrition intervention study. At baseline there was no statistical difference in the mean retinol of both groups. Anaemic participants in the control group (anaemic-control) had mean serum retinol concentration of 23.78 ± 5.23 μg/dl whilst the anaemic participants in the supplemetal group (anaemic-supplemental) had mean retinol concentration of 27.46 ± 7.28 μg/dl at the end of the study. The anaemic-control had prevalence of low vitamin A as 64.3% at baseline whilst the anaemic-supplemental had it as 84.2% (Table 4). At end-line, low vitamin A prevalence was 23.1% in the anaemic-control and 21.1% in the anaemic-supplemental. Between group difference of difference in low vitamin A status was 21.9% (Table 4), statistically significant, *p* = 0.046. The anaemic-control had mean haemoglobin concentration of 106.6 ± 5.1 g/l at baseline whilst anaemic-supplemental had mean haemoglobin concentration of 104.9 ± 8.2 g/l. At the end-line, the anaemic-control had mean haemoglobin concentration of 105.7 ± 7.5 g/l and the supplemental had it as 113.6 ± 13.6 g/l (Table 4). The prevalence of anaemia among the anaemic-control was 46.3% at baseline. It was 53.7% in the anaemic-supplemental. At the end of the study prevalence of anaemia was 34.1% in the anaemic-control and 29.3% (Table 4) in the anaemic-supplemental. The results (Table 5) showed household income, infection status, stunting status and intervention diet were the factors linked to the anaemia status of the participants in this study. Thus non-consumption of composite green leafy vegetables powder was significantly and negatively associated with anaemia, *p* < 0.05. Malaria parasitaemia was prevalent among the children in both groups. There was no significant difference in the level of malaria parasitaemia in the participants in the control and the supplemental groups at the beginning and at end-line.

**Table 1:** Baseline and household characteristics of study participants

Factor	Treatment			
	Control (*n* = 51)	CGLVP (*n* = 53)	Difference	*P*-value
	(Mean ± SD)	(Mean ± SD)		
Age (years)	7.3 ± 1.7	6.7 ± 1.8	0.6	0.081
Gender (%)				
Boys	51.0	50.9	0.1	0.765
Girls	49.0	49.1	0.1	0.761
Anthropometric indices				
Weight (kg)	21.6 ± 3.9	22.9 ± 4.9	1.4	0.113
Height (cm)	117.1 ± 11.3	120.6 ± 11.1	3.6	0.110
Weight-for-age z score	−0.485 ± 0.92	−0.731 ± 0.91	0.246	0.193
Height-for-age z score	−0.722 ± 1.15	−0.59 2 ± 1.17	0.130	0.567
Body Mass Index (kg/m^2^)	−0.388 ± 0.92	−0.176 ± 0.91	0.212	0.134
Stunting (%)	17.6	13.2	4.4	0.530
Wasting (%)	15.7	11.3	4.4	0.514
Thinness (%)	3.9	5.7	1.8	0.281
Overweight (%)	3.9	3.8	0.1	0.689
Malaria parasitaemia (%)	37.3	34.0	3.3	0.726
Mosquito net use (%)				
Yes	(86.3)	(90.6)		0.491
No	(13.7)	(9.4)		
Beneficiary of National Health Insurance Scheme (%)				
Yes	88.3	83.0		0.452
No	11.7	17.0		
Parental education (%)				
Formal education	94.1	96.2		0.712
Informal education	5.9	3.8		
Parental occupation (%)				
Formal sector	11.8	1.9		0.110
Informal sector	88.2	98.1		

*P*-values significant at *p* < 0.05 using student t-test, otherwise chi-square test

**Table 2:** Mean haemoglobin levels and prevalence of anaemia among study participants

Factor	Treatment			
	Control	CGLVP	Difference	*P*-value*
	n (Mean ± SD)	n (Mean ± SD)		
Haemoglobin concentration (g/l)				
Baseline	51 (116.9 ± 9.9)	53 (117.6 ± 12.7)	0.7 ± 2.5	0.747
End-line	47 (113.4 ± 8.5)	51 (121.9 ± 13.5)	8.5 ± 4.5	0.001
Difference	−3.5 ± 1.4	4.3 ± 0.8	7.8^a^	0.028
*P*-value^1^	0.160	0.026	–	–
Effectiveness (%)	−3.0	3.7	6.7	–
Prevalence of anaemia (haemoglobin conc. < 115 g/l), (%)				
Baseline	37.3	41.5	4.2	0.657
End-line	57.5	33.3	24.2	0.024
Difference	−20.2	8.2	28.4^a^	0.011
*P*-value^1^	0.294	0.030	–	–
Effectiveness (%)	−54.2	19.8	74	–

*P*-values significant at 0.05 using student-t test otherwise chi-square test

Negative values in table shows reduction in prevalence or means

*P-value for between group comparison

^1^*P*-value for within group comparison

^a^Difference in difference values

**Table 3:** Serum retinol levels and prevalence of vitamin A deficiency among study participants

Factor	Treatment			
	Control	CGLVP	Diff^a^	*P*-value*
	n (Mean ± SD)	n (Mean ± SD)		
Serum retinol concentration (μg/dl)				
Baseline	46 (16.79 ± 8.74)	50 (16.97 ± 7.74)	0.18 ± 1.01	0.907
End-line	44 (24.35 ± 5.50)	46 (26.96 ± 6.86)	2.61 ± 1.36	0.065
Difference	6.57 ± 1.74	9.18 ± 1.57	2.43^a^	0.067
*P*-value^1^	0.001	0.0001	–	–
Effectiveness (%)	39.1	54.1	15.0	–
Low vitamin A status (serum retinol conc. < 20 μg/dl) (%)				
Baseline	60.9	66.0	5.1	0.062
End-line	15.9	13	−2.9	0.084
Difference	−45.0	−53.0	−8.0^a^	0.056
Effectiveness (%)	−73.9	−80.3	−6.4	–
Adequate vitamin A status (serum retinol conc. 20- < 50 μg/dl) (%)				
Baseline	40.0	34.0	−6.0	0.058
End-line	84.0	87.0	3.0	0.081
Difference	44.0	53.0	9.0^a^	0.053
Effectiveness (%)	104.5	125.8	21.3	–

*P*-values significant at 0.05 using student-t test otherwise chi-square test

Negative values in table shows reduction in prevalence or means

*P-value for between group comparison

^1^*P*-value for within group comparison

^a^Difference in difference values

**Table 4:** Post vitamin A and anaemia status of participants anaemic before the intervention study

Factor	Treatment			
	Control (*n* = 19)	CGLVP (*n*-22)	Diff^a^	*P*-value*
	n (Mean ± SD)	n (Mean ± SD)		
Serum retinol concentration (μg/dl)				
Baseline	19 (16.63 ± 7.59)	22 (14.39 ± 5.40)	−2.24 ± 1.19	0.457
End-line	18 (23.78 ± 5.23)	13 (27.46 ± 7.28)	3.68 ± 2.05	0.089
Difference	7.15 ± 2.36	13.07 ± 1.88	5.92 ± 0.48^a^	0.047
*P*-value^1^	0.001	0.0001	–	–
Effectiveness (%)	43.0	90.8	47.8	–
Prevalence of low vitamin A (serum retinol conc. < 20 μg/dl) (%)				
Baseline	64.3	84.2	19.9	0.049
End-line	23.1	21.1	−2.0	0.892
Difference	−41.2	−63.1	−21.9^a^	0.041
Effectiveness (%)	−64.1	−74.9	−10.8	–
Haemoglobin concentration (g/l)				
Baseline	19 (106.6 ± 5.1)	22 (104.9 ± 8.2)	−1.7 ± 3.1	0.450
End-line	14 (105.7 ± 7.5)	12 (113.6 ± 13.6)	7.9 ± 6.1	0.046
Difference	−0.9 ± 2.4	8.7 ± 5.4	9.6 ± 3.0^a^	0.034
*P*-value^1^	0.489	0.011	–	–
Effectiveness (%)	−0.8	8.3	9.1	–
Prevalence of anaemia (Hb < 115 g/l) (%), *N* = 41				
Baseline	19 (46.3)	22 (53.7)	7.4	.062
End-line	14 (34.1)	12 (29.3)	−4.8	0.081
Difference	−12.2	−24.4	− 12.2^a^	0.042
Effectiveness (%)	−26.3	−45.3	−19.0	–

*P*-values significant at 0.05 using student-t test otherwise chi-square test

Negative values in table shows reduction in prevalence or means

*P-value for between group comparison

^1^*P*-value for within group comparison

^a^Difference in difference values

**Table 5:** Factors associated with anaemia among the study participants

Factor	Odd Ratio	95% CI	*P*-value
Age	0.9	0.75–1.3	0.719
Gender			
Boys	0.7	0.27–1.7	0.461
Girls	1	Reference	
Household Income			
≤ GHC 500	2.5	0.25–24.2	0.440
>GHC500	1	Reference	
Infection Status			
Malaria Presence	1.4	0.59–3.5	0.421
Malaria Absence	1	Reference	
Stunted			
Yes(HAZ˂ -2 SD)	1.2	0.34–4.4	0.751
No(HAZ˃ -2 SD)	1	Reference	
Intervention diet			
No powder	2.8	1.1–6.9	0.036
Powder	1	Reference	

Statistical significance set at *p*< 0.05, child age was adjusted for, *OR* Odd Ratio

## Discussion

This is one of few studies done with green leafy vegetables in Ghana [29, 30] and the first in the study area that investigated the effect of composite leafy vegetable powder on the anaemia and vitamin A status of school children. The baseline data showed prevalence of anaemia (39.4%) and low vitamin A levels (63.5%) among the children. The present findings of the study like previous studies across various regions of Ghana had showed that prevalence of anaemia among school aged children in the southern and northern sectors of Ghana is of public health interest [3, 4, 31–33]. Anaemia is attributable to the influence of nutritional and anti-nutritional factors [10, 11]. The current study showed infection (malaria parasitaemia) existed among the children and they consumed mostly plant based diets made of staple cereals and legumes. These are rich sources of anti-nutritional factors such as polyphenols, tannins and phytates, known inhibitors of iron and zinc absorption and bioavailability [13]. The prevalence of vitamin-A deficiency among the children in the current study is above the level reported previously [4]. The analyzed data showed that the mean haemoglobin concentration of the children who consumed Composite Green Leafy Vegetable Powder (CGLVP) increased significantly over that of those who consumed only the normal stew and soup provided by the school feeding program. There was a 3.5 g/l decline in the mean haemoglobin concentration within the control group whilst the mean haemoglobin concentration increased by 4.3 g/l within the experimental group. Previous studies demonstrated that vitamin A supplementation or fortification in controlled intervention studies increased the haemoglobin concentrations of preschool children [32]. The present study demonstrated that dietary modification of stew and soup with CGLVP; provitamin A and nonhaeme iron rich green leafy vegetable powder increased haemoglobin concentration of school aged children. The findings demonstrated that consumption of CGLVP minimized the prevalence of anaemia by 8.2% among the participants whilst consumption of the normal stews and soup provided by the school feeding program resulted in 20.2% rise in the prevalence of anaemia among the study participants. At the end of the study, the findings indicated that prevalence of anaemia was significantly higher among the control group compared to the supplemental group. Mean serum retinol concentration improved significantly within the two study groups. The likely consumption of palm fruit products at the household level aside the school lunch meal might have caused the significant increase in the mean retinol concentrations of both study groups. The later part of the study period coincided with the onset of palm fruit harvest. These intakes were confounders beyond the control of the intervention program. CGLVP consumption increased the mean serum retinol concentration of study participants by 2.4 μg/dl over the mean serum retinol concentration of the control group but this was not statistically significant, *p* = 0.067. The consumption of CGLVP was 15% more effective in improving mean serum retinol concentration than non-consumption of CGLVP among the participants. Before the start of the nutrition intervention, both the control and CGLVP groups had mean serum retinol concentration (17.0 μg/dl), considered as marginal vitamin A status (serum retinol concentration 10 – 20 μg/dl), [18]. The current study findings agree with suggestions made in earlier studies [34, 35] that intervention strategies targeted at reducing both vitamin A and iron deficiencies would be more effective in reducing anaemia compared to those aimed at either of these micronutrients. The powder was 19% more effective in minimizing prevalence of anaemia among the anaemic supplemental participants compared to the anaemic control in the course of the intervention study. The mean change (difference of difference), in serum retinol concentration was 5.92 ± 0.48 μg/dl for the anaemic participants. The composite leafy vegetable powder (CGLVP) stews and soup were 47.8% effective in causing this mean change in serum retinol concentration between the study participants in the two groups. The consumption of the CGLVP (supplemental) stews and soup lead to 21.9% change (otherwise difference in difference) in prevalence of low vitamin-A status among the anaemic participants in the study. The CGLVP stews and soup were 11.0% effective in minimizing prevalence of low vitamin-A status among the anaemic participants. The public health significance of vitamin A deficiency (VAD) would be classified as severe at baseline, since the findings show that at least 20% of the study participants had vitamin A deficiency [18]. The baseline dietary data (not presented in results of the current paper) indicated minimal dietary diversity among the children. They consumed less meat and meat products, less dairy products, moderate fruits and vegetables. Previous studies provided a lot of evidence that linked vitamin A deficiency to less frequent consumption of vegetables, fruits, fish, meat and less varied household diet [36, 37]. Under normal physiological conditions serum retinol concentration reflects body vitamin A status only when liver retinol stores are seriously depleted. Inflammation affects serum retinol concentration. Severe infections also cause a decrease in vitamin A intake which reflects low vitamin A status [38]. At the end of the study prevalence of low vitamin A status (serum retinol concentration < 20 μg/dl) reduced greatly in both study groups but 3.8% more in the intervention group. The findings indicated that improvement in vitamin A status of participants in both study groups (24.35–26.96 μg/dl) resulting in 18.2 and 18.8% VAD in the control and intervention groups. That was an indication that VAD would be categorized as moderate prevalence in terms of public health significance [18]. The addition of groundnut oil or coconut oil to the stews and soup promoted dietary fat intake of participants in both study groups. Dietary fat intake is known to improve bioavailability of dietary carotene [39, 40]. Consumption of CGLVP plus vegetable (groundnut or coconut oil) oil was 15% effective in reducing prevalence of low vitamin A status than normal stews and soup provided by the school feeding program. CGLVP consumption was also 21.3% more effective in promoting adequate vitamin A status (serum retinol concentration 20 - < 50 μg/dl) among the participants. Non-consumption of CGLVP was strongly and significantly associated with anaemia among the children. Participants who did not consume CGLVP had 2.8 times higher risk of being anaemic compared to participants who consumed CGLVP. The findings demonstrated that household income was strongly associated with anaemia even though this association was not significant. Children who belonged to households with monthly income less than or equal to GHȼ500.00 had a higher risk (2.5 times) of being anaemic than children who belonged to households with monthly income more than GHȼ500.00. A previous study reported that consumption of nutrient rich foods among rural poor folks was inversely proportional to food prices [41] and probably directly related to income levels. Most often the income factor could be associated with the nutritional knowledge of the individual, household head and the home maker. The most significant factor associated with anaemia in this study was the intervention diet. Study participants who did not consume CGLVP had 2.8 times higher risk of becoming anaemic than children who consumed CGLVP.

### Limitation(s) to the study

The study participants were children benefiting from the Ghana School Feeding Program. For this reason the findings could not be applied to children in non-school feeding program schools and also children not attending school. Dietary intake of palm fruit products at household levels towards the end of study could not be controlled and hence their influence might be a confounding factor to vitamin A status of the participants.

## Conclusion

The consumption of composite green leafy vegetable powder increased the mean haemoglobin concentration significantly but not the mean serum vitamin A concentration of the study participants. It minimized the prevalence of anaemia among the participants. Composite green leafy vegetable powder has the potential to minimize prevalence of anaemia among the study participants.

## Abbreviations

CGLVP: Composite green leafy vegetable powder
g/l: gram per litre
GLVs: Green leafy vegetables
HPLC: High performance liquid chromatography
IQ: Intelligence quotient
NMIMR: Noguchi Memorial Institute for Medical Research
RDAs: Recommended dietary allowances
VAD: Vitamin A deficiency
